# Medical physics practice in the next decade

**DOI:** 10.4103/0971-6203.28017

**Published:** 2006

**Authors:** Bhudatt Paliwal

**Affiliations:** 600 Highland Avenue, K4/B100, Madison, WI 53792, USA

**Keywords:** Adaptive radiotherapy, breathing synchronized

## Abstract

Impressive advances in computers and materials science have fueled a broad-based confluence of basic science breakthroughs. These advances are making us reformulate our learning, teaching and credentialing methodologies and research and development frontiers. We are now in the age of molecular medicine. In the entire field of health care, a paradigm shift from population-based solutions to individual specific care is taking place. These trends are reshaping the practice of medical physics. In this short presentation, examples are given to illustrate developments in image-guided intensity-modulated and adaptive helical tomotherapy, enhanced application of intensity modulation radiotherapy (IMRT) using adaptive radiotherapy and conformal avoidance. These advances include improved normal tissue sparing and permit dose reconstruction and verification, thereby allowing significant biologically effective dose escalation and reduced radiation toxicity. The intrinsic capability of helical TomoTherapy for megavoltage CT imaging for IMRT image-guidance is also discussed. Finally developments in motion management are described.

It is a great pleasure and honor to be invited to deliver the Dr. Ramaiah Naidu oration. In the early thirties of the twentieth century, Dr. Naidu established the foundations of medical physics. He was a post-doctoral fellow of Madame Curie and was the first medical physicist at the Tata Memorial Hospital, Bombay, where he installed a Radon production facility. He implemented a similar facility at the Sloan Kettering Memorial Institute in New York. If he were here today, he would be amazed how the field has changed. Table 1 lists some of the major landmarks.

**Table 1:** Summary of major landmarks

*1895 - 1950 Age of discovery*	*1950 – 2006 Evolution of technology*	*Imaging tools*
Discovery of X-rays (Röentgen)	Medical linac US (Ginzton)	Orthogonal X-rays
140kV X-ray tube (Coolidge)	Use of cobalt-60	Fluoroscopic sim
Linear acceleration principle (Ising)	SW linac (Knapp)	CT, DRR, CT/sim
Proof of principle (Wideröe)	Exploration of high energies	Ultrasound
Microwave cavity theory	Computer treatment planning	MR, PET, SPECT
Rhumbatron oscillator (Hansen)	Multileaf collimation	EPID
Magnetron (Randall and Boot)	3D conformal therapy	Radiocamera systems
Definition of radiation units	Intensity modulation radiotherapy, tomotherapy	Optical systems
Basic principles of treatment	Image guided radiotherapy	On board imaging
Medical linac UK (MRC)	Particle therapy: Proton, light ions	Cone beam and 4DCT

4DCT - Four-dimensional computed tomography, PET - Positron emission tomography, SPECT - Single photon emission computed tomography, EPID - Electronic portal imaging device

During the last decade, the complexity of detection, planning and delivery tools for medical services has increased exponentially. The computer age has been a double-edged sword. It has significantly enhanced our capabilities while also increasing the potential for greater risks in the delivery of our services. New technology not only requires a highly skilled medical physicist - it also requires continuous updating of knowledge and skills and integration of new technological developments in our practice. There are new challenges in all of the following areas: Learning, teaching, research and development and clinical services.

## Challenge in learning

In the past the pace of change was slow and we used to become a qualified individual for life. In the current age of rapid change, we will need to be responsive to these changes and to be accountable to the high expectation of our clients (patients). The competencies required in the practice of clinical medical physics have grown: a professional medical physicist needs communication, managerial and scholastic competence and should also be a collaborator and advocate. To meet these obligations, training and education we receive to qualify as a certified medical physicist would require a continuous process of maintenance of certificate. It would need to be responsive to change. It would need to be an educational initiative for continuous personal development (CPD), an activity designed to enhance knowledge, skills, attitudes and competencies, required for practice. It would need to be documented and have measurable outcomes from engaging in CPD. The overall goal would be to improve practice performance, enhance the quality of patient care, safety. Moreover, nowadays all the scientific fields, similar to medical physics, are approaching a multidisciplinary paradigm. In order to keep up with the development of any field, an individual has to learn the latest changes in technology and other related skills, viz., imaging sciences, algorithm design, or use of soft computing techniques.

## Challenge in teaching

Our knowledge base in the last decade has increased dramatically (The American Association of Physicists in Medicine report # 79) and continues to expand significantly. How and what we teach impacts many health sciences professionals. We teach medical physicists, radiation therapists, radiation oncologists and radiologists. Unfortunately our teaching methods have not changed and are archaic. We need to develop clinically relevant teaching tools, incorporate modern web-based technology and use interactive processes to teach clinically relevant material that reflects professional needs. The American Association of Physicists in Medicine (AAPM), American Board of Radiology (ABR) and American Society for Therapeutic Radiation Oncology (ASTRO) are all developing new syllabi and teaching tools. Some specific challenges we face are:

- Retaining highly regarded faculty
- Declining federal funding for research support
- Large class sizes
- Realigning to the new era of medicine with focus on molecular biology and genomics
- Imaging to emphasize function and biological interactions, micro-imaging and pinpoint therapy
- Space needs for larger training programs

In the long run, with these efforts we may claim to have trained better health sciences professionals based on higher board scores or decreased mistakes; however, the real differences can be measured only in the quality of care delivered to patients.

## Challenges in research and development

Significant physical and biochemical developments in the radiation oncology related fields have created potential for dose escalation in the delivery of radiation treatments. Some of these developments are based upon new compounds and models and many are from new imaging tools shown in Table 1. These tools allow higher confidence in tumor targeting and normal tissue sparing by providing reliable mechanisms for generation and delivery of conformal dose distributions. There are significant advances in localizing the target as a function of time, particularly four-dimensional computed tomography (4D-CT). These advances allow us to shift the isodose distribution to the surface of the target volume with a rapid fall-off in the normal tissue. Several changes have been, in part, technologically mediated and to some extent due to the synergy between, and integration of, radiotherapy and other modalities. These processes are likely to continue in the next decade. Some of the major impacts will come from the following.

## Radiobiological modeling

During the last 30 years, radiobiological modeling has evolved on many frontiers. The concepts of nominal standard dose[1,2] gave way to mean nominal tumor dose[3] and equivalent uniform dose (EUD)[4] and generalized equivalent uniform dose (gEUD).[5] The linear quadratic model came into prominence, and modern statistics-driven modeling tools have been outstanding in formulating new fractionation schemes. Modeling lessons have established that dose escalation improves local control, but treatment lengthening retards the gain. Tomé and Fowler[6] suggest the feasibility of increasing effects by boosting 60-80% of the target by boost-dose ratios of 1.2 to 1.3. There are few situations where a boost-dose ratio exceeding 1.3 appears to be worthwhile or necessary. Significant increases of tumor control probability, up from 50% to 75%, might be achieved for a small increase in risk of necrosis, where a substantial proportion of the target volume (60-80%) can be boosted.

## Imaging advances

**Figure 1:**
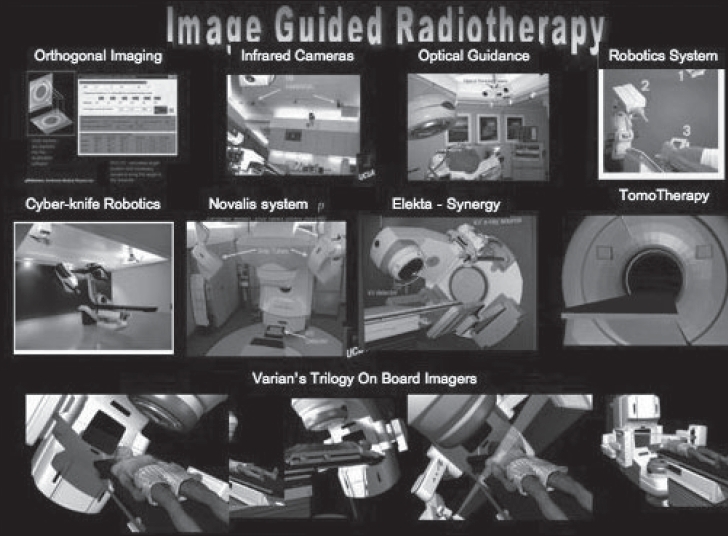
Modern imaging system incorporated delivery systems

The need for safe delivery of inhomogeneous dose distributions without increasing risks to normal structures has provided an impetus to the development of imaging tools coupled to radiation delivery systems. Examples of some of these are given in Table 1 and Figure 1. These range from conventional orthogonal radiographic systems to state-of-the-art TomoTherapy, cone beam computed tomography (CT), CT-PET (positron emission tomography) and magnetic resonance imaging (MRI) systems.

**Figure 2:**
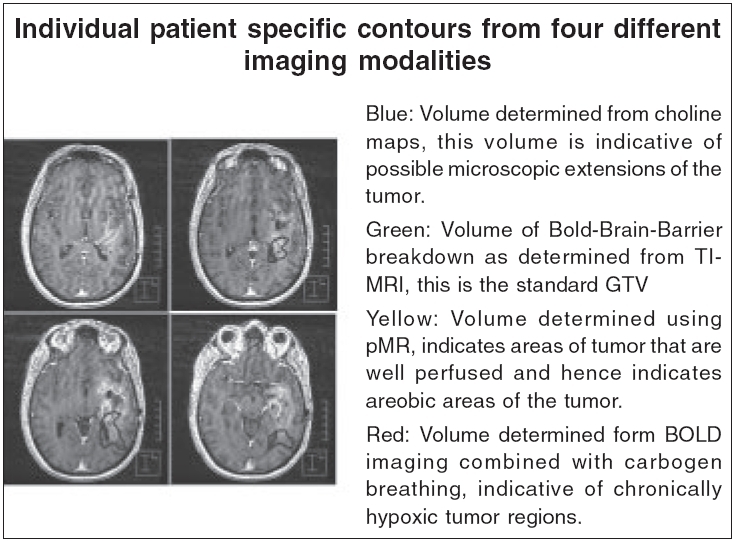
Multimodality imaging based region of interest delineation

Molecular imaging is a rapidly evolving area, with a focus on monitoring gene expression *in vivo* using remote imaging devices (positron emission tomography [PET], single photon-emission computed tomography [SPECT], MRI, optical and ultrasound). Molecular imaging as now envisioned actually exploits creative synergies between these hierarchies of signal sources, resulting in fusions between these biological scales. Figure 2 shows incorporation of target definition boundaries determined using four different tissue properties in brain images. The blue contour is the volume determined from choline maps; this volume is indicative of possible microscopic extensions of the tumor. The green outline is the volume of Blood-Brain-Barrier breakdown as determined from T1-MRI; this is the standard gross target volume (GTV). The yellow outline shows the volume determined using percutaneous myocardial revascularization, indicating areas of tumor that are well perfused and hence indicates aerobic areas of the tumor. Lastly, the red contour, determined from blood oxygenation level dependent imaging combined with carbogen breathing, is indicative of chronically hypoxic tumor regions. All of these, to a degree, are important factors to be considered in the determination of the target volume, its radiation sensitivity and the prognosis of outcome.

## Technology-driven delivery methods

Technological developments in computers and accelerator designs, particularly inverse treatment planning and dynamic multi-leaf collimation systems (dMLCs), have given the radiotherapy community the ability to deliver conformal and intensity-modulated radiation therapy (IMRT) treatments. With the implementation of image-guided radiation therapy (IGRT), there is a potential[6] to deliver inhomogeneous dose distributions that increase the dose inside the target by 20-30% over the minimal peripheral dose, while decreasing possible normal tissue complications by collapsing the planning target volume (PTV) onto the clinical target volume (CTV), as is often done in stereotactic radiosurgery.

## Image-guided intensity-modulated and adaptive helical TomoTherapy

Image-guided IMRT is redefining the practice of radiation oncology. Traditional methods of implementing beam intensity modulation have included individually designed compensators, static multi-leaf collimators (MLC), dynamic MLC and sequential (serial) tomotherapy. Helical TomoTherapy[7] provides added functionality to enhance the application of IMRT. It facilitates adaptive radiotherapy and conformal avoidance. These advances improve normal tissue sparing and permit dose reconstruction and verification, thereby allowing significant biologically effective dose escalation and reduced radiation toxicity. Recent radiobiological findings can be translated into altered fractionation schemes that aim to improve the local control and long-term survival. The intrinsic capability of helical TomoTherapy for megavoltage CT (MVCT) imaging for IMRT image-guidance is an added feature aiding in further adaptation of treatments.

## Helical TomoTherapy

**Figure 3:**
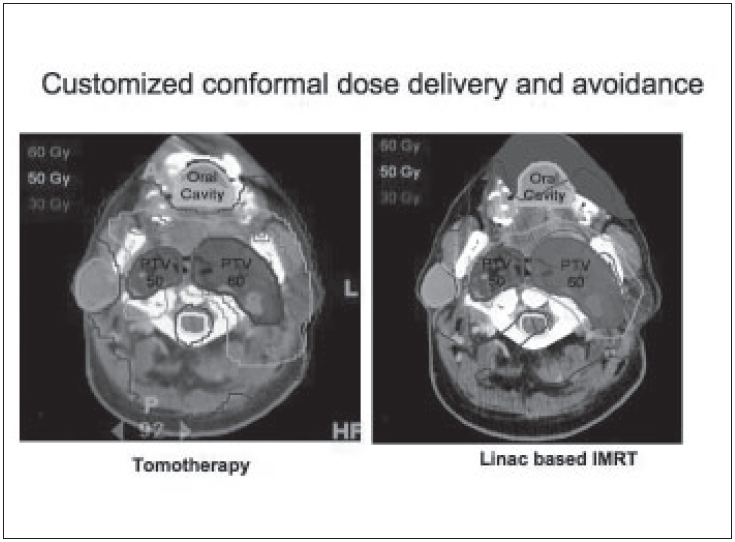
Image-guided conformal dose delivery and avoidance

In contrast to standard radiotherapy, helical TomoTherapy [Figure 1] delivers treatment with a rotating intensity-modulated fan beam. The patient is continuously translated through a ring gantry, resulting in a helical source trajectory about the patient. The beam delivery is similar to that of helical (‘spiral’) computed tomography (CT) and requires slip rings to transmit power and data. The ring gantry provides a stable and accurate platform to perform tomographic verification of both the patient setup and delivered dose. The design of the helical TomoTherapy unit allows for continuous delivery over 360 degree beam angles.[8] The helical delivery minimizes the risk of significant high or low dose deposition in areas of overlap or junctioning.[9] Assessments of sequential units presently in use reveal that positioning errors as little as 1 mm can cause dose errors of the order of 10-20% in the abutment regions.[10] In addition to full integration of IMRT delivery, an important advance with helical TomoTherapy over the other current systems is the ability to provide accurate verification of radiation delivery via onboard tomographic imaging. Examples of conformal avoidance planning and delivery using a Linac and Tomotherpay unit are given Figure 3.

## Adaptive radiotherapy

**Figure 4:**
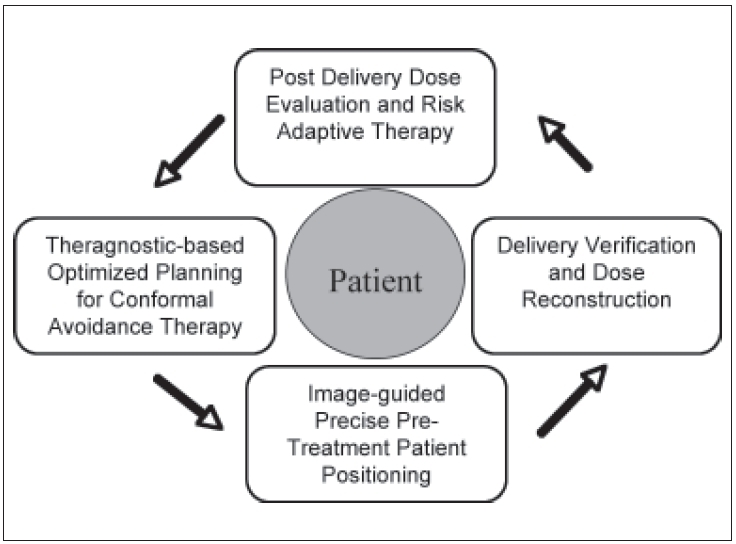
Major steps in modern radiotherapy

One of the significant features of the helical TomoTherapy unit is the presence of an integrated online MVCT unit. This permits verification of patient positioning, target tumor/organ registration to assess internal motion (including geometric shift and shape/volume changes) and reconstruction of delivered dose. These capabilities offer the radiation oncology team the ability to verify and adjust the therapeutic plan as needed during the course of treatment. This concept is referred to as adaptive radiotherapy.[11] These capabilities can be viewed as a closed-circuit loop, as illustrated in Figure 4. The integration of the MVCT and the treatment unit allows for options not possible with contemporary systems. For example, if a patient setup is found to differ from the planned position, the current approach requires moving the patient to compensate for this positioning error. With the integrated helical unit, another option is having the patient remain in the ‘incorrect’ position and modifying the treatment delivery. The success of the modification is independent of the extent and direction of the offsets, within certain limits.[12] MVCT images can be obtained at radiation doses of around 2 cGy and are comparable to that of diagnostic CT imaging[13,14] and lower than reported doses from low-dose megavoltage cone beam CT.[15] Other methods of onboard imaging have been developed recently and are available clinically.

## Dose reconstruction

Newly developed dose reconstruction tools use data acquired during the delivery of a treatment to determine the actual three-dimensional dose deposited. At the time of treatment on the TomoTherapy unit, the incident energy fluence is computed from the signal detected at the exit detectors. An accurate, anatomically detailed 3D representation of the patient is also obtained. A transfer matrix then converts this signal to incident energy fluence. In other words, the matrix determines the energy fluence emitted from the MLC from the signal at the detector. The integrated CT detectors present in the TomoTherapy unit provide detailed information about the primary projection, as well as the scatter characteristics of every projection. Pathlength and detector-to-patient distance are computed from the MVCT image. Leakage and transmission plus tongue and groove penumbra are also included in the calculation. This fluence corrected for some additional MLC effects is used in the treatment dose distribution computation. Effectively, this provides an accurate daily delivered dose record, which can be compared directly with the planned dose distribution.

## Dose comparison

**Figure 5:**
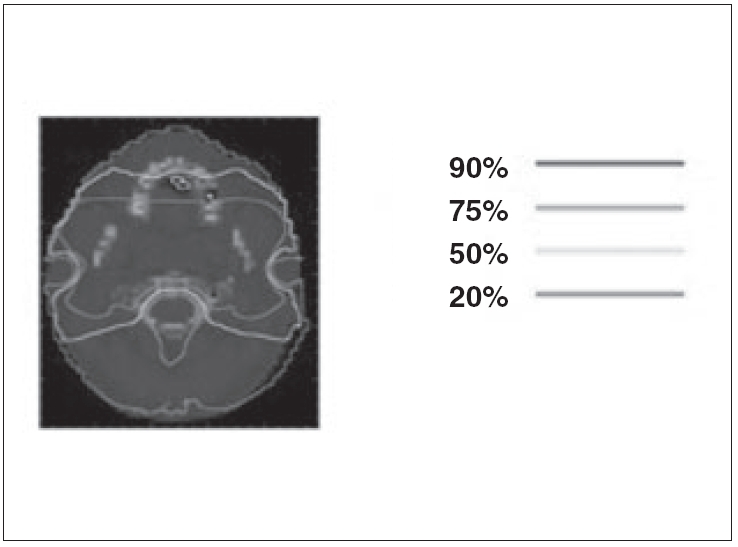
An example of helical tomotherapy optimization used for a nasopharyngeal case. The primary tumor and the regional fields are identified as targets. The parotid glands and spinal cord were considered avoidance regions and, thus, the dose to these structures was minimized to lower than 50% of the primary dose

The ability to accurately reconstruct the 3D dose distribution permits a quantitative comparison of desired and actual dose distributions delivered to the patient. An example illustrating this issue is described in Figure 5. Desired isodose lines of a helical TomoTherapy optimization for a nasopharyngeal cancer are depicted with the parotid glands and spinal cord areas to be avoided.

The comparison of desired dose distributions to the actual delivered dose distributions is based on the methods of Van Dyk *et al*.[16] and Low *et al*.[17] The two modes of comparison are dose difference (DD) and distance-to-agreement (DTA) analyses. For regions in which both the planned and measured distributions have high dose gradients, DTA comparisons are conducted. For all other cases, DD analyses are performed. Once the mode of comparison is decided, the × index is computed by dividing the DD and DTA values by their respective tolerances.

The × value provides a measurement of quality for every voxel, indicating if they are within the desired tolerance or how far they deviate from that tolerance. Typical tolerance values in IMRT are 3% and 3 mm for DD and DTA respectively. The smaller the × value, the more accurately the compared isodose distributions are aligned. To facilitate the spatial identification of problem areas, color-wash images of × maps can be displayed [Figure 5a–d]. Figure 7a, b illustrates the × image comparing the planned dose and the reconstructed dose for the first week of treatment in which a systematic error was made. Several error regions appear on the targets and regions at risk, mainly in the high gradient regions. Figure 7b shows a gray scale image of the re-optimized dose distribution that could be delivered during the second week of treatment in order to correct for prior misalignment. In this dose image, a pattern appears that is very similar to the × image.

**Figure 6:**
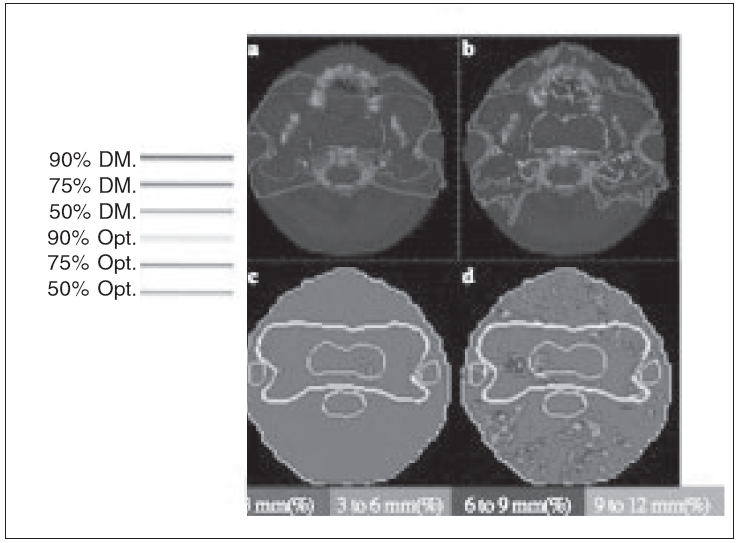
Examples of (a) successful and (b) unsuccessful delivery modifications following treatment given with a displacement from the intended patient position. The corresponding ξ figures are shown in (c) and (d) for the successful and unsuccessful delivery respectively. Red indicates that the dose distributions are between 0 and 3% or 0 and 3 mm (within tolerance), green between 3 and 6% or 3 and 6 mm, blue between 6 and 9% or 6 and 9 mm and light blue >9% or >9 mm

**Figure 7:**
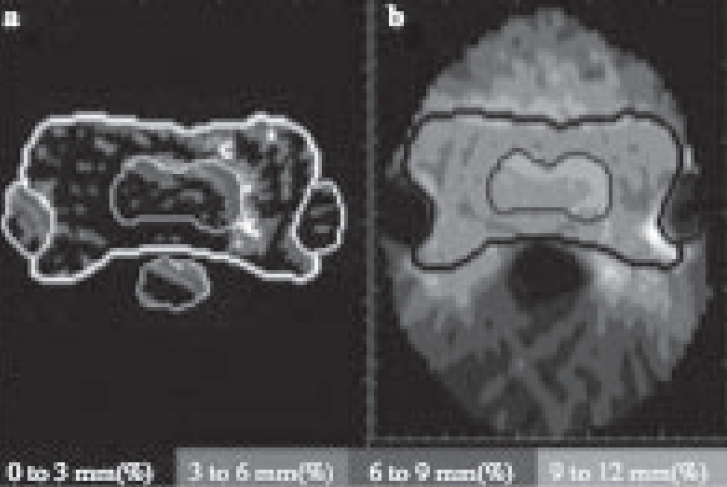
Example of optimization to correct for one week of incorrectly delivered dose. Panel ‘a’ is a ξ image of one week of delivery with the patient shifted by 0.5 cm in the × and 0.5 cm in the y direction. Panel ‘b’ is the gray scale image of the dose to be delivered in the second week, designed to compensate for the previous week’s error

**Figure 8:**
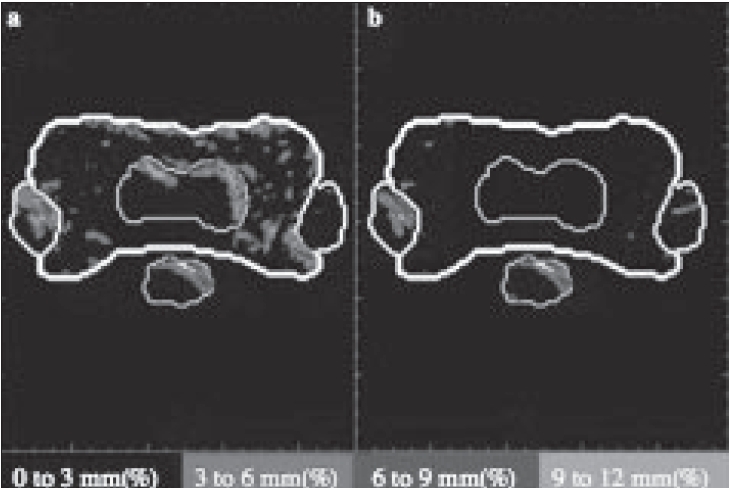
Panel ‘a’ compares the ξ image after the second week if no action is taken to correct for errors during the first week. Panel ‘b’ shows the ξ image if action is taken to correct for the error

**Figure 9:**
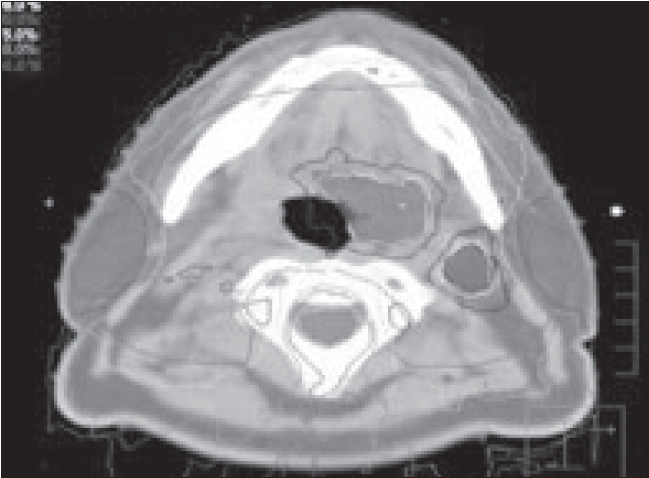
Conformal avoidance tomotherapy. The tumor target (red) and the grossly involved node (blue) are contoured and planned for high-dose conformal therapy, whereas the parotids (purple) are simultaneously conformally avoided

Figure 8 illustrates the result of one week of treatment with a systematic error followed by a second week of either the original treatment plan given accurately (Figure 8a) or a re-optimized treatment plan designed to compensate for the errors of the first week (Figure 8b). Simply repositioning the patient and accurately delivering treatment in the subsequent week according to the originally designed radiation plan will dilute the error incurred during the first week but cannot fully compensate for the error. Radiation delivery modifications are designed to compensate for the difference between actual and desired dose distributions. In the example presented, dose reconstruction and comparison reveal that excess dose is being deposited in the spinal cord and right parotid. In Figure 9 a modified plan specifically designed to compensate for the errors of week one is instituted in the second week and the dose reconstruction is performed again. Most of the errors within the tumor region are corrected. However, a small trade-off with dose to the parotid glands and spinal cord is necessary in order to rectify the errors incurred during the first week of delivery. The specific thresholds for the trade-offs that should be accepted remain a matter of ongoing physics and clinical research.

With helical TomoTherapy, MVCT images can be registered using a full mutual information algorithm, bone extracted feature fusion (EFF) and bone and tissue EFF algorithms, with uniform down-sampling of the MVCT images along the x and z axes (to provide a time-saving by a factor of up to 4) with and without the rotational registration components. These particular algorithms take into account any changes in patient anatomy between the reference image and fusion image when the image registration is carried out.

## Conformal dose-per-fraction escalation

The advances in adaptive radiotherapy, conformal avoidance and enhanced localization and immobilization have provided an impetus for novel time dose fractionation dose delivery schemes to improve local control and possibly increase survival rates. These dose-fractionation regimens are target specific (lung, prostate, etc.) and provide significant sparing of normal tissues, increase the total dose and dose per fraction, and reduce the number of fractions delivered to reduce the overall treatment time.

## Motion management

Many IMRT approaches rely on increasing the number of beam directions and modulating beam intensity such that multiple tiny sub-beams are created. While this improves dose distributions, the clinical applicability of such pencil beams requires immense precision; the slightest patient/organ/tumor motion is likely to result in unintended errors. Therefore, although highly irregular dose distributions and dose volume histograms (DVHs) can be created, their clinical application is uncertain. Some of the patient immobilization and motion management systems to address this issue are described below.

## Optical guidance

**Figure 10:**
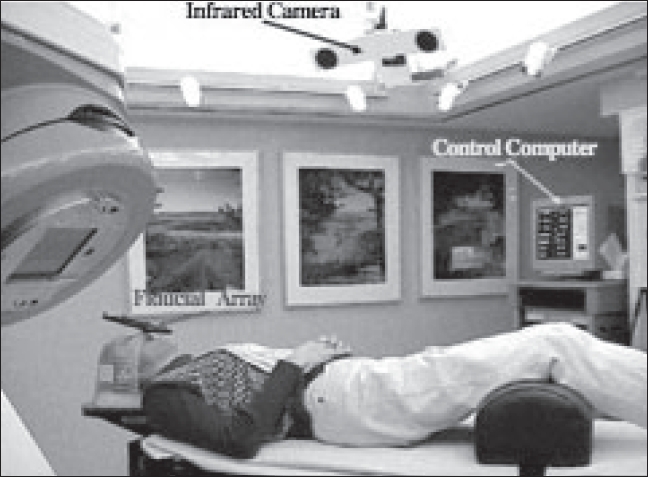
Layout of infrared cameras and fiducial arrays in a treatment room. Translations and rotations are tracked in real time using the optical position-sensor systems. These systems allow one to localize patients between fractions in the treatment room within 0.3 mm translation error and 0.3 degrees of rotation error

Noninvasive head frame systems often rely on external contours of the head and face and have immobilization errors of 2-4 mm in favorable circumstances. Such systems include the Heidelberg system, which has a reported accuracy of 2 mm;[18] the Laitinen’s stereoadapter with a measured error of approximately 2-3 mm;[19] and the Gill-Thomas-Cosman, which uses a maxillary bite block system to yield reproducible immobilization and repeat fixation[20] with a reported accuracy of 0.5-1 mm. The optically guided FSRT/IMRT approach in use at the University of Wisconsin is a noninvasive system shown in Figure 10, where localization is separated from immobilization. This is accomplished through detection of four markers attached to a custom rigid bite plate. The location of these markers in space (tracked relative to the isocenter) is accomplished in real time using an optical position-sensor system mounted to the ceiling of the accelerator vault and interfaced with a computer. The interfraction translational error and rotational error is within 0.3 mm and 0.3 degrees respectively. Tomé *et al*.[19–20] have shown that this optically guided system, in conjunction with IMRT planning, allows the generation of highly conformal treatment plans that exhibit smaller 90, 70 and 50% prescription isodose volumes, improved PITV ratios (the ratio of the prescription isodose volume to tumor volume), comparable or improved EUD, smaller NTDmean for critical structures and an inhomogeneity index that is within generally accepted limits. In addition, optically guided treatments allow real-time monitoring of treatment delivery, providing further confidence in the patient’s actual delivered dose distribution. For helical TomoTherapy, such optical guidance will be useful in verifying that the patient has not moved between MVCT and treatment.

**Figure 11:**
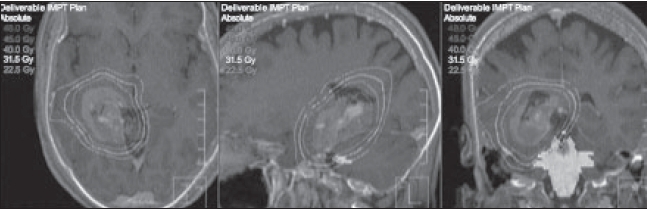
A pilocytic astrocytoma treated to a total dose of 45 Gy with treatment planning performed using three approaches: a standard 3-field, an FSRT and a helical tomotherapy IMRT approach

**Figure 12:**
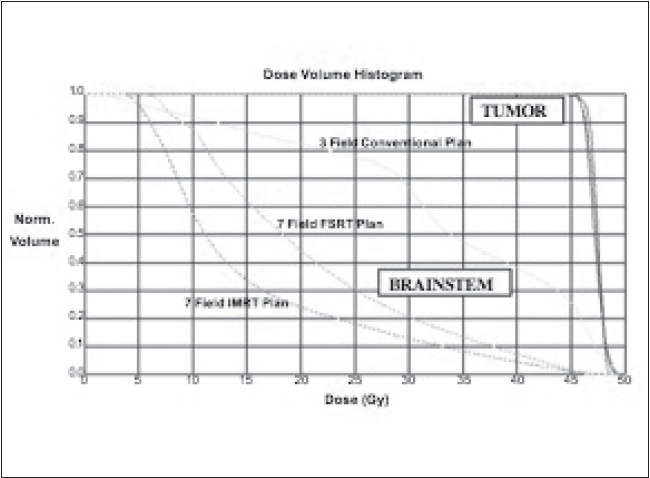
DVH comparisons of the three plans shown in Figure 11

To demonstrate the clinical relevance of optical guidance, an early implementation of this system is illustrated in Figures 11 and 12. This patient with an astrocytoma required radiotherapy and was planned with 3-D conformal techniques, multi-non-coplanar field FSRT and IMRT. In all three treatment planning scenarios, CT-MR fusion was utilized and the defined GTV/CTV was constant. A mathematical descriptor PITV (the ratio of the prescription isodose volume to tumor volume) is frequently employed to evaluate dose conformality. The ideal PITV ratio is 1; values up to 2 are commensurate with good stereotactic radiosurgery plans. In the example presented in Figure 11, the PITV values are 3.16, 1.65 and 1.45 for the 3-field conventional, 3-D FSRT and helical TomoTherapy plans respectively. The DVHs in Figure 12 reveal substantial improvement in brain stem dose reduction as the technical approach becomes more sophisticated. The superior patient immobilization, day-to-day alignment and position-verification afforded by the FSRT and the IMRT systems using an optically guided system allowed for a substantial reduction in the PTV margins.[20] This margin reduction alone can have a significant impact in improving the DVH. The conformality afforded by FSRT and IMRT leads to dosimetric improvement that might be further improved using helical TomoTherapy techniques. The application of immobilization devices is practical, and the incorporation of optical guidance provides a high degree of reliability in terms of daily positional reproducibility and for monitoring intra-treatment motion, potentially maximizing the inherent benefits of helical TomoTherapy.

## Ultrasound guidance

The system described above works well as long as the rigid body approximation holds, as is the case for intracranial lesions. However, outside the cranium, soft-tissue targets can move relative to rigid fixation points (e.g., bony structures) between the times of image acquisition, treatment planning and treatment delivery. Real time imaging is useful in determining target location at the time of treatment delivery. A system based on 3D-ultrasound guidance (SonArrayTM, ZMed, Inc., Ashland, MA) can be used to correct for these misalignments at the time of treatment. Ultrasound is chosen because it is a flexible and inexpensive imaging modality that can easily be adapted for use in a radiation therapy treatment room. The interpretation of two-dimensional ultrasound images can be challenging and is highly dependent on the skill of the operator in manipulating the transducer and mentally transforming the 2D images into a 3D structure. Three-dimensional ultrasound imaging overcomes this limitation. The 3D ultrasound data sets are generated through optical tracking of free-hand acquired 2D ultrasound images. The position and angulation of the ultrasound probe are determined using an array of four infrared light-emitting diodes (IRLEDs) attached to the probe. An infrared camera is used to determine the positions of the IRLEDs and this information is input to the computer workstation. The position of each image plane can therefore be determined using the IRLEDs and an ultrasound volume can be reconstructed by coupling the position information with the images. In addition to building the 3D image volume, optical guidance is used to determine the absolute position of the ultrasound image volume in the treatment room coordinate system. Because the relative positions of the 3D-image volume and the ultrasound are fixed, knowledge of the probe position in the treatment room coordinate system at the time of image acquisition is sufficient to determine the position of the image volume relative to the linac isocenter. The image-to-probe relationship is determined by a calibration step performed at the time of system installation.[21] In this way, ultrasound guidance will allow greater accuracy of treatment delivery via helical TomoTherapy to various extracranial sites.

Currently, ultrasound is being used in conjunction with MVCT. We are presently conducting a comparison of ultrasound with MVCT. Ultimately, it may be that MVCT alone will be the only image guidance necessary.

## Respiratory gating

Two strategies have emerged to deal with the problem of respiratory motion. One strategy is to ‘immobilize’ the lung during one phase of respiration and to gate radiation to this phase. This requires the ability of patients to hold breath for a short period of time, which may be difficult in patients with respiratory cancers. The second strategy is to radiate at a predetermined period during respiration using dynamic aperture tracking. Online verification of the correct phase of respiration requires a respiratory monitoring device.[22] Another option provided by the helical TomoTherapy unit is to determine the phase of respiration using the MVCT. Individual patient respiratory patterns will be assessed during planning, and an MVCT can be performed at each treatment. The treatment will then be gated to the predicted respiratory phase with the appropriate dosimetric plan.

## Motion management

**Figure 13:**
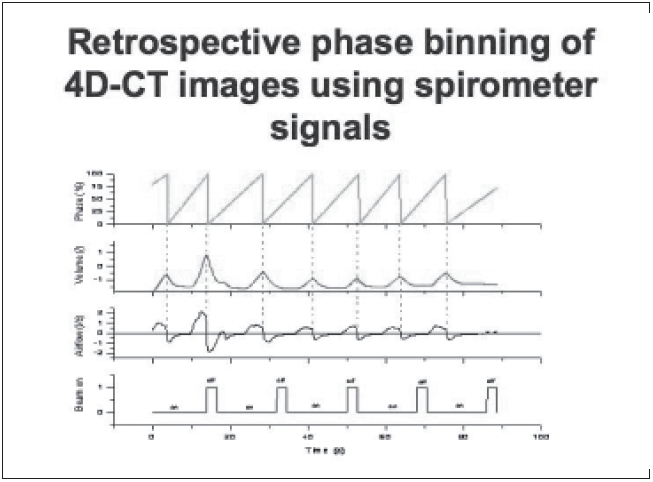
Retrospective phase binning of 4D-CT images using spirometer signals. The 0% phase is defined as the full inhalation phase and is detected at the positions where the airflow has zero values and a negative slope (indicated by dotted lines)

**Figure 14:**
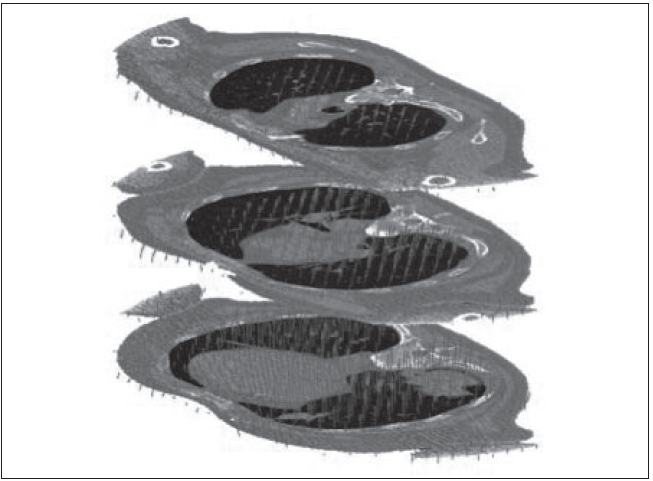
Deformable registration

**Figure 15:**
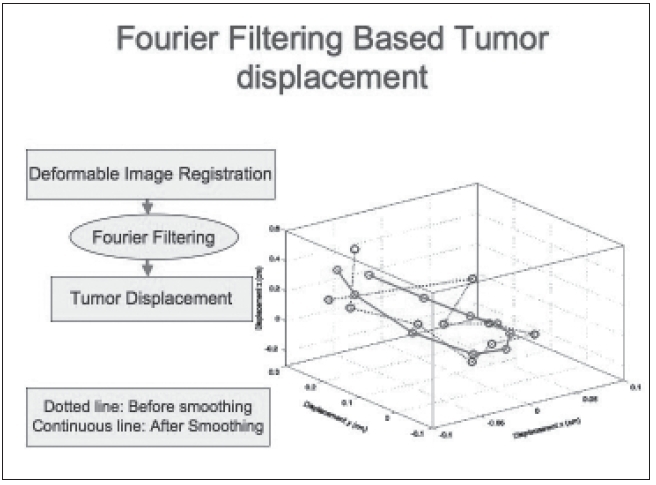
The average trajectory of the target obtained by deformable image registration and smoothed by Fourier filtering. (Dotted lines: before smoothing; and solid line: after smoothing). The major component of displacement is in z direction (superior-inferior)

**Figure 16:**
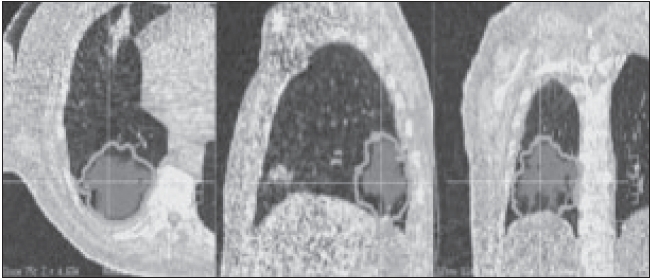
Motion envelope derived by deformable image registration. The area in color wash is the PTVex (original PTV excluding breathing motion). Contours show the envelope on the treatment planning CT images (at full exhalation phase)

**Figure 17:**
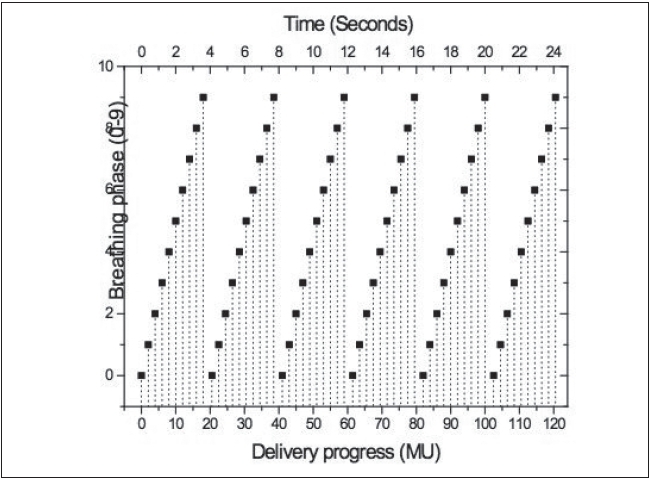
The correlation of breathing phases and delivery process. Assuming periodic breathing pattern and constant dose rate, the breathing/delivery phase correlation is set prior to treatment planning

**Figure 18:**
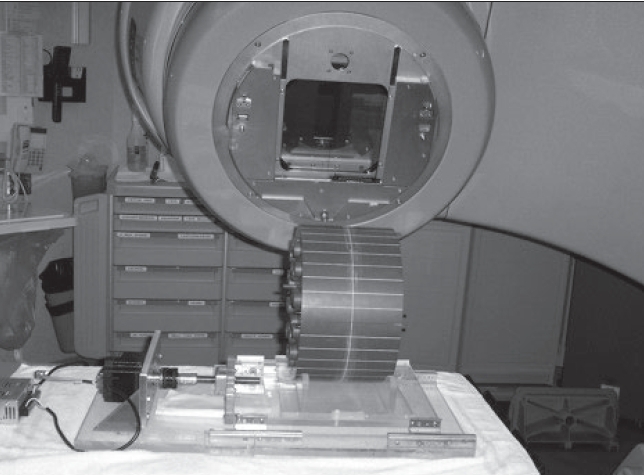
The mobile phantom used in dosimetry study. The cheese phantom is driven by stepper motor and custom-developed software. It can simulate the actual linear motion pattern recovered from 4D-CT images

**Figure 19:**
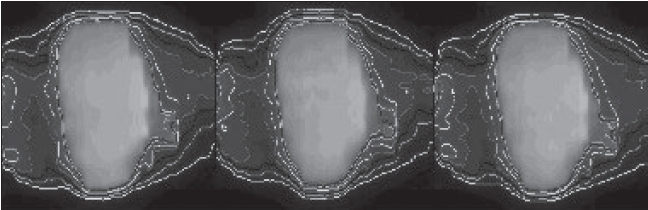
Exposed film and relative dose distribution. Left: conventional plan delivered on static phantom; Middle: conventional plan delivered on moving phantom; Right: BSD plan delivered on moving phantom

**Figure 20:**
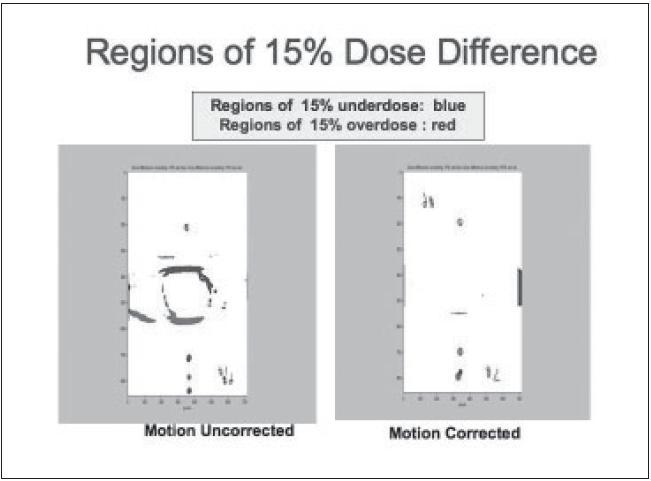
Dose difference exceeding -20% are in blue and 20% are in red

An attempt is made here to give an overview of some of the recent developments. The concepts of motion synchronization of treatment delivery will be elaborated. During the last 5 years, there has been significant development in breathing-synchronized delivery (BSD) of 4-D optimized IMRT treatment plans.[23–35] Due to space limitations, a snapshot of some of the completed work is given in Figures 13–20.

In BSD, patient breathing and treatment delivery are synchronized by instructing the patient to breathe following a breathing guide. We have previously developed a 4-D TomoTherapy treatment technique based on BSD. We have extended the application of BSD into conventional radiation treatment using dMLCs. Dynamic 4D-CT images are acquired for treatment planning, and the images at full exhalation phase are used as treatment planning images. A typical IMRT treatment plan is developed that yields dynamic leaf sequences for sliding window delivery of IMRT. Average target trajectory is obtained by deformable image registration of 4-D data sets and is smoothed by Fourier filtering. Assuming the patient breathes with a reproducible breathing pattern and a constant dose delivery rate, the treatment process is corre-lated with the breathing phase in BSD treatment planning. Figures 13–17 show the correlation of breathing phases, deformable registration, motion envelope multileaf collimation generation and delivery processes. The instantaneous target displacement is overlaid to the dMLC position at the corresponding phase.

A custom-built mobile [Figure 18] phantom was used in dosimetry verification. The phantom, driven by a computer-controlled stepper motor, is able to move according to the linear pattern used in BSD treatment planning. The conventional plan is delivered on the phantom with and without motion. The BSD plan was also delivered on the phantom that moved with the prescheduled pattern and synchro-nized with the delivery of each beam. Film dosimetry results [Figures 19 and 20] revealed that without incorporating motion, underdose and overdose (over 20%) were noticed at the superior and inferior regions of the target respectively. BSD delivery, on the other hand, obtained dose distribution very similar to that planned. No region in the target had dose delivery error of more than 20%, as planned.

The BSD technique described for use with dMLC is similar to the method of real-time tracking. This method basically needs no hard-ware modification and avoids the difficulties of real-time tracking. Unlike cardiac motion, breathing motion, to some extent, can be voluntarily controlled by some patients. Studies have shown that many patients may be able to maintain a regular breathing pattern with some instruction. We are currently developing a patient train-ing device to allow more practice for the patient. Since BSD allows continuous air exchange, it is better tolerated than breath-hold gating.

We realize that breathing motion is an extremely challenging frontier in the delivery of precision radiotherapy and is a function of several physiological, behav-ioral and mechanical processes. Even though we may have developed an idealized 4D-CT/PET and patient-guiding cycle based on optimized IMRT treatment, its delivery is significantly susceptible to the above-mentioned processes during the delivery of the plan. The baseline filling of the lung has also been identified as a significant factor in the determination of the daily position of the target volume, which makes daily pretreatment imaging indispensable. We believe that further refinement of the BSD technique will be possible using the on-board imaging verification processes offered by TomoTherapy and kilovoltage cone-beam CT.

## Conclusion

**Figure 21:**
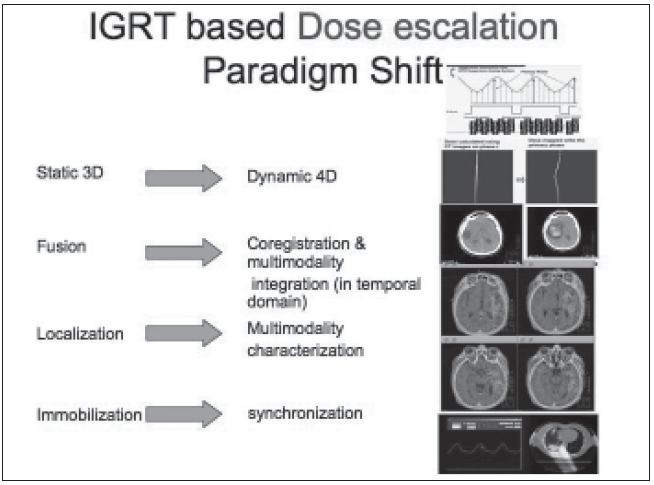
IGRT-based dose delivery paradigm shift in radiation therapy

The availability of new imaging modalities before and during radiotherapy planning and delivery has allowed us to launch new tools at every major step of the treatment process. Figure 21 shows that instead of a 3-D static patient data set, we need to incorporate dynamic 4-D patient data and corresponding beam warping to prepare a motion-compensated treatment plan. Instead of fusion of data from merely two modalities, tumors need to be characterized and localized using multimodality imaging-based information. Instead of merely delivering treatments with external patient immobilization, we need to achieve internal motion synchronized beam delivery.

Radiation therapy is unique among the cancer treatment modalities because it can be modulated in the four dimensions of time and space. Modulation in the time domain gives rise to the time-dose-fractionation problem, and this continues to be one of the most fruitful avenues for further improvement of the therapeutic ratio in radiation oncology. Spatial modulation has been greatly facilitated by recent technological advances in image guidance based treatment planning and delivery. Progress in functional and molecular imaging, together with an improved understanding of the patterns of spread of cancer, provides the tools that will allow us to prescribe 4D radiation therapy that is optimized in the individual patient. All of these developments, combined with progress in biological and chemical targeting of cancer cells, provide not only challenging and exciting research opportunities but also a real hope that more cancer patients will be cured (or left with a manageable chronic disease in place of a lethal one).

Between-patient differences can be identified and exploited with ever-greater finesse by ever-finer levels of an ever-increasing inventory of covariates, which now include an increasing number of biological and biochemical measures on continuous scales, as well as the coarser nominal and ordinal demographic measures (e.g., stage, gender, etc.) of the past.

**Figure 22:**
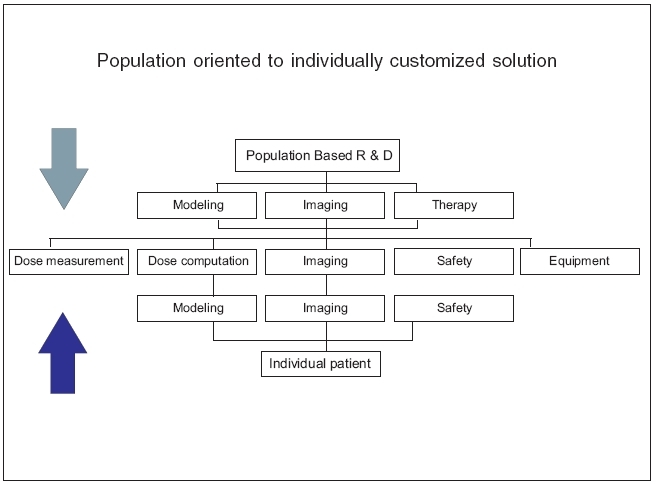
Population to individual customization of therapy

Moreover, within-patient differences across time, e.g., the circadian variations in patient physiology and the infra-treatment organ motion that continually scans the target volume across the steep dose gradients of the IMRT radiation fields during each daily treatment can now be tracked and their effects either exploited or compensated for: Modern (‘adaptive’) radiotherapy can now routinely ‘hit a moving target.’ All of this diversity of information is very patient specific, and we are challenged to seek individual patient-specific solutions [Figure 22] instead of the prior practice of mere population-based approaches.

Clinical implementation of IMRT, especially image-guided IMRT, is in a state of rapid evolution. Helical TomoTherapy, one the latest steps in this evolution, is an IMRT system whose design incorporates aspects such as infinite beam angle optimization and MVCT-based delivery verification. These features have the potential to permit the full clinical development of adaptive radiotherapy and conformal avoidance. To realize the ultimate goal of improving clinical outcomes for our patients, appropriate patient immobilization, optimized target localization, conformal avoidance of sensitive normal structures and radiobiologically-guided dose escalation are required. The clinical implementation of helical TomoTherapy and the consequent issues raised (such as radiation dose rates and dose homogeneity) present questions and opportunities that may change the current paradigm in radiation oncology.
